# From Acceptable Profit to Fair Return: Operational Benefit-Sharing Models for Induced Pluripotent Stem Cell Research Using Legacy Samples from Low- and Middle-Income Countries

**DOI:** 10.21203/rs.3.rs-10411560/v1

**Published:** 2026-08-03

**Authors:** Clement A. Adebamowo, Peter A. Ikhane, Temilola Yusuf, Sally N. Adebamowo

**Affiliations:** University of Maryland School of Medicine; University of Maryland School of Medicine; Center for Bioethics and Research; University of Maryland School of Medicine

**Keywords:** benefit sharing, biobanking, induced pluripotent stem cells, commercialization, low- and middle-income countries, community engagement, research ethics, global health governance, Nigeria

## Abstract

**Background.:**

There has been no previous study that specifies how benefit sharing could be delivered for biospecimens from low- and middle-income countries (LMICs) that enter high-value translational uses such as induced pluripotent stem cell (iPSC) research. Stakeholders endorse benefit sharing in principle, but the mechanisms that would make it deliverable, the actors who would guarantee it and the benefits that would count as fair remain undeveloped and contested and are further complicated by sample anonymization and by commercialization that crosses borders.

**Methods.:**

We conducted a framework analysis of qualitative data from the KACIY study, concerning the proposed reuse of stored, immortalized HapMap Project samples from the Yoruba community of Ibadan (YRI) for iPSC research. Data comprised key-informant interviews with six community advisory board (CAB) members and five United States genomic-research bioethicists, and two focus group discussions with twenty community members. We coded and charted the data against five axes: profit legitimacy, benefit types, delivery agents, timing of agreement, and feasibility constraints, and compared responses across the three stakeholder groups. Reporting follows the COREQ guidance.

**Results.:**

We found that participants treated commercial profit as legitimate but treated benefit sharing as necessary to prevent extractive research and to preserve trust, and that they distinguished one-time compensation from ongoing benefit. Preferred benefits formed recurring bundles, which were subsidized access to resulting medicines and products, community infrastructure, capacity building and local co-investigation, recognition, and, for a minority, a proportional share of profits. Proposed guarantors included the national government and Ministry of Health, international bodies such as the World Health Organization, an independent third-party fiduciary, repositories acting through material transfer agreements, and local researchers and the CAB. Support for government custodianship was qualified by distrust. Bioethicists raised most of the feasibility constraints, which were funder rules, anonymization and cross-border enforcement. Participants held that terms should be agreed at the start of a project and that commercialization should be stated in the consent.

**Conclusions.:**

We translate this support into an architecture that can be implemented, comprising a tiered benefit-bundle menu with a guaranteed minimum and negotiated additions, an accountable guarantor model with escalation and an independent complaint channel, and contractual insertion points in material transfer agreements, data return requirements and product access commitments. Our study shows that benefit sharing for LMIC samples is best approached as a problem of implementation, and it specifies mechanisms that keep benefit sharing feasible, explicit and protective of community trust while avoiding the inflation of expectations.

## Background

Biobanks turn a single sample donation into a durable resource for secondary use. For example, samples collected from the Yoruba community of Ibadan, Nigeria, for the International HapMap Project in 2001 were anonymized, immortalized and stored, and have since supported genome-wide association studies (GWAS) and a generation of population genomic discovery [1, 2]. Two decades later, induced pluripotent stem cell (iPSC) technology makes it possible to reprogram these samples into living cell lines that can become many tissue types, which opens a high-value translational pathway that did not exist when the samples were collected [3]. We previously reported that stakeholders connected to the YRI sampling context regard iPSC use of these anonymized, broadly consented samples as ethically workable when broad consent is combined with sustained community engagement, transparent governance and practical mechanisms for reciprocal benefit [1].

Benefit sharing is an opportunity for researchers to engage with participants in truly reciprocal relationships. It is particularly salient for LMIC samples entering high-value pipelines, because the interval between donation and reuse widens the asymmetry between the communities that made the science possible and the institutions and companies that are positioned to profit from it, and because African genomics has a documented history of extractive research [4, 5]. This is not because of lack of principles or guidance. The Human Genome Organization issued a Statement on Benefit-Sharing in 2000, which grounded obligations in common heritage, distributive justice and solidarity [6]; the Nagoya Protocol and the CIOMS guidelines set out fair and equitable sharing of benefits arising from the use of genetic resources [7, 8]; and African communitarian accounts derive reciprocity, solidarity and accountability as governing values for genomics [4]. What has been lacking is implementation. A study of genomics researchers in sub-Saharan Africa found support for benefit sharing on grounds of fairness, solidarity and reciprocity, but little awareness of what it entails and a lack of clarity about how it could be implemented in practice [5].

This concern is not abstract for genomics research in Africa. Repeated episodes in which samples and data left the continent with little return have hardened into a widely shared narrative of extraction and have made benefit sharing a test of whether researchers treat African communities as partners or as sources of raw material [5, 4, 9]. iPSC research raises the stakes further, because reprogrammed cell lines are not a static dataset but a renewable, high-value input to therapeutics, organoids, and drug-screening platforms whose commercial trajectories are long and global [3, 10]. Previously, we identified elapsed time as a distinct determinant: the two decades between donation and proposed iPSC reuse widen both the technological gap the community must be helped to understand and the value gap it is asked to accept without return [1]. Benefit sharing is the mechanism through which that widening gap is meant to be closed.

Three features of the YRI iPSC case make the implementation problem acute. First, the samples are anonymized, so individual donors cannot be re-contacted, and individual compensation is not available, and any return must reach a community rather than a person. Second, iPSC products may be commercialized through global markets far downstream of the donor community, so the mechanism of return must survive distance, time and multiple intermediaries. Third, stakeholders distinguish one-time compensation from ongoing benefit, a distinction that has direct policy consequences and that existing frameworks rarely operationalize. In this paper, we move from establishing that stakeholders support benefit sharing to specifying how it could be delivered. We asked which benefit bundles are perceived as fair and how they are justified, who is trusted to guarantee benefits and on what basis, and how stakeholders separate benefit sharing from compensation. We then translate the answers into a benefit-sharing architecture for LMIC samples used in iPSC research, which can be extended to other types of research.

## Methods

### Design

We conducted qualitative analysis of data generated by the “Knowledge, Attitude and Recommendations of Community members, Researchers, and Bioethicists on creation of iPSC from YRI HapMap samples (KACIY) study”, which examined stakeholder perspectives on broad consent and on the reuse of HapMap YRI samples for iPSC research [1]. The present analysis addresses benefit sharing. Our reporting follows the Consolidated Criteria for Reporting Qualitative Research (COREQ-32) [11], and a completed checklist accompanies the submission.

### Setting and context

The source community for the HapMap YRI samples is Ibadan, Oyo State, Nigeria. The YRI samples were donated anonymously, immortalized and stored at the Coriell Institute (New Jersey, United States), from which cell lines are distributed under material transfer agreements to researchers throughout the world. The prompting question for KACIY was whether these samples could ethically be reprogrammed for iPSC research, a use unforeseen at collection [1, 3].

### Participants and sampling

We collected data between November 2023 and May 2024 from three purposively sampled stakeholder groups. We enrolled six of the eight members of the community advisory board (CAB) constituted for the HapMap project for key informant interviews (KIIs); one member was unavailable and one was deceased. Their ages ranged from 76 to 87 years (mean 83.0, SD 4.36), two were women and four were men, five were Christian and one was Muslim, and five had retired while one was self-employed. We purposively recruited twenty members of the community where the YRI samples were collected for two focus group discussions (FGDs) of ten participants each, and we balanced participants by sex, age, religion and socio-economic status. Their ages ranged from 18 to 77 years (mean 44.2, SD 15.6), eleven were women (55%) and nine were men (45%), eleven were Christian (55%) and nine were Muslim (45%), and nine were traders, five were professionals, two were students, two were retired and two were stay-at-home or unemployed. We enrolled five bioethicists for KIIs, four of whom had been involved in enrolling participants at the YRI site and one of whom was the principal investigator of the repository where the samples are banked. All five were women; three were university professors of whom two had retired, one was a retired researcher at the National Institutes of Health, and one worked in a research institute in the United States. This triangulated design allows us to compare how benefit-sharing expectations are expressed by those whose communities are the source of the samples, by those who represent them and by those who steward the resource professionally.

### Data collection

We developed KII and FGD guides organized by constructs drawn from published literature on the principles of research ethics and on the ethics of iPSC research. The guides covered benefit sharing, commercialization, samples and data sharing, ownership and intellectual property, among other topics. We piloted the guides with three individuals outside the study community and modified and finalized them on the basis of the pilot. Because we were concerned that CAB and community members may have had limited knowledge of iPSC, we prepared an information sheet on iPSC from publicly available sources and shared it with them before the study began. We conducted the CAB KIIs in offices within the community, or virtually, or in hybrid format, and each lasted approximately one hour. We conducted the two FGDs in the community, and each lasted one hour. We conducted the bioethicist KIIs in person or virtually, and each lasted 60 to 90 minutes. P.A.I. led all interviews and focus groups. T.Y. was the notetaker for the CAB KIIs and the FGDs, and C.A.A. was the note-taker for the bioethicist KIIs and attended the CAB KIIs virtually without participating. All interviews and focus groups were audio-recorded, and we recorded additional notes on participants’ behavior and body language. Each participant gave informed consent. The numbers of CAB members and bioethicists were fixed by their prior participation in the HapMap project, and we judged that twenty FGD participants would be sufficient to reach data saturation given the homogeneity of the study population.

### Data management and analysis

We transcribed the interviews using a combination of a speech-to-text transcriber (Otter.ai) and manual methods, and we reviewed the transcripts for clarity and correctness. In the parent study we conducted two independent analyses of the KII and FGD data using a combination of manual and computer-aided qualitative data analysis with NVivo. We created a priori codes from our research objective as a general framework while allowing for emergent concepts, wrote a description of each code in the code manager, grouped codes into families according to shared themes, and created semantic networks representing hierarchical relationships between concepts. For the present analysis we applied the Framework method to the transcripts, the analytic memos and the de-identified excerpts from the previously published study [1]. We structured an initial coding matrix around five *a priori* axes derived from our research questions, which are profit legitimacy, benefit types, delivery agents or intermediaries, timing of agreement and feasibility constraints, and we supplemented these with inductive codes where the data exceeded the frame. One analyst coded the material, and a second reader reviewed the coding, and we resolved discrepancies by returning to the transcript. We charted coded segments into a stakeholder-by-axis matrix to support systematic comparison across the three groups, and from this we developed the benefit-bundle typology and the guarantor model. We actively sought disconfirming cases, in particular feasibility skepticism and distrust of proposed guarantors. Because the focus group format makes individual attribution unreliable, we report the range and patterning of views across groups rather than counts of individuals. We identify participants by group and code, as CAB, P3 to P8; FGD, R1 to R20; and BE, P1 to P5.

### Reflexivity

Members of the analytic team are investigators on the parent study and have a long-standing relationship of trust with the source community, particularly with the lead investigator. This relationship shaped what participants said and how we interpreted it; in the benefit-sharing context it may have encouraged optimistic accounts of what researchers can deliver. We mitigated this by anchoring every claim to verbatim language, by attending to feasibility skepticism voiced by bioethicists, and by explicitly separating stakeholder preferences from any commitment by a sponsor or repository.

## Results

We report findings along the five axes and then compare the three stakeholder groups. Community and CAB participants reasoned in concrete terms about medicines, buildings and jobs, while bioethicists reasoned about structures, feasibility and enforcement. The two registers are complementary and together specify a workable architecture.

### Commercial profit is accepted as legitimate

Participants in all three groups treated commercialization as a normal and acceptable part of translational research rather than as something illicit. Community and CAB participants reasoned that investors and companies must recoup their costs and that profit sustains further discovery.

*“They will (should) make money because they have to sell the products that they produce from it (samples). So, they have to be compensated for the research.”* (CAB, P6)

*“The sponsors rendered help in sponsoring the research, so they should get something back.”* (FGD, R15)

*“It is good to make a profit; it will enable them to move forward.”* (FGD, R9)

Bioethicists observed that willingness to accept commercialization is itself conditional on a credible account of return; being told how a community benefits is, on this view, part of what makes commercial research trustworthy rather than extractive.

*“Not just trust, but I’ll trust you more if you can tell me how we or my people or I benefit or my family benefits.”* (BE, P4)

Participants did not objectt to profit. They objected to the selling of the samples themselves, as distinct from products derived from them, and to the absence of any return. Bioethicists shared the acceptance of commercialization and identified the equity problem that arises when returns bypass the donor community.

*“if therapies or tests or treatments are developed, yeah, companies that develop this will make a lot of money from it, and that money is not going to the people in Nigeria who give the samples.”* (BE, P3)

### From acceptable profit to expected return: reciprocity, not charity

Acceptance of profit was accompanied by an expectation of return, which participants framed as reciprocity and as a safeguard against being used and then forgotten. A community member described the breach that a failure to reciprocate would represent.

*“you shouldn’t engage people in research and then completely forget about them.”* (CAB, P5)

Bioethicists gave the same expectation a policy shape, insisting that benefit must exceed one-off payment and explicitly separating compensation from benefit, and rejecting the older position that a contribution to the general good of science is a sufficient return in an LMIC community context.

*“it’s no longer acceptable just to say you will not benefit from this research, but you’ll contribute to the broader good of society… We’re really talking about separating compensation from benefits… it needs to be more than that… if you’re working in a low to middle income country… it has to be more than whatever money you gave to the participants.”* (BE, P2)

Community members made the same distinction from the other direction, tying return to the moment profit is realized and defaulting to the community where individual records are unavailable, which is the operational consequence of anonymization.

*“The sample donors are supposed to be compensated if you have their records but if not, their community should be considered… the sample donors should be compensated whenever there is profit.”* (FGD, R12)

### What benefits count: recurring benefit bundles

Preferred benefits clustered into recurring types rather than a single demand. We label these bundles because participants typically endorsed several at once and treated them as substitutable to some degree.

#### Subsidized access to resulting products and medicines.

The most frequently named benefit was affordable access to the medicines and therapies that the research might yield, delivered to the donor and participating communities, with the subsidy financed by the research itself.

*“The drugs produced from such research should be taken to donor/research-participating communities at a subsidized rate. The subsidy should be funded by the research.”* (FGD, R4)

*“Sizable benefits (e.g. medications) should go to participating sites (communities).”* (FGD, R6)

The justification offered for subsidized access was distributive: participants framed affordable, locally reachable products as the point of the whole enterprise, contrasting them with therapies that exist but remain out of reach for the poor or require travel abroad.

*“We are aware that (organ) transplantation is very costly, and not many people can afford it. If you can use the samples to provide solutions that will be within easy reach of poor people, this will be wonderful… it will still be better than flying people to Germany or India for treatment.”* (FGD, R12)

For some, the value of access was concrete and personal, grounded in their own dependence on medicines that reliable supply makes possible.

*“it is good because I am asthmatic, and it is the medication that I take that has been helping me.”* (CAB, P3)

#### Community infrastructure and development.

Participants pointed to durable community assets, citing a hall the HapMap project had provided as a concrete precedent, and, recurrently, health facilities.

*“The benefits can come through community development programs such as the hall donated to the Aba-Alamu community by the HapMap project.”* (FGD, R9)

Some located responsibility for such assets with the state, envisaging government provision of the facilities and materials that translational research requires, a theme we return to when considering guarantors.

*“the government should provide all the materials; all the necessary infrastructure to do this kind of work.”* (CAB, P6)

#### Capacity building, local co-investigation, and recognition.

Bioethicists emphasized non-monetary returns that build lasting capability and acknowledge the community’s contribution, echoing the earliest benefit-sharing statements [5].

*“we have to be honest in benefit sharing… the community that helps you build the map should be recognized that they’re on the map, thanked for being on the map and make sure that the research… involves those communities and their researchers as well.”* (BE, P1)

One bioethicist connected these expectations to the earliest institutional benefit-sharing efforts, recalling direct involvement in framing recognition and capacity building as constitutive elements of benefit rather than optional extras [6].

*“we included it in recognition and thanking the community. We included it in capacity building.”* (BE, P1)

#### A proportional share of returns.

A minority proposed an explicit, weighted profit share that treats the community’s contribution as a stake in the value created, a notably sophisticated model from a community participant.

*“this source will get a 2.5%, a 5%, or 1.5%, whichever way… if you left the research process to technology alone… they will never get anything out of it without the contribution of the people. So, you just have to weight it (the contributions of those involve) and build that.”* (CAB, P5)

Direct individual payment was the least emphasized and the most constrained, because anonymization removes the records that individual compensation would require; participants consistently redirected individual entitlement to the community as the standing beneficiary [1, 12].

### Who guarantees benefits: candidate intermediaries and their legitimacy

Participants regarded researchers as poorly placed to move money, and proposed a range of guarantors, each with a distinct claim to legitimacy and distinct liabilities.

#### An independent third party.

The clearest articulation held that benefit delivery is not the researchers’ competence and requires a dedicated, equity-oriented intermediary.

*“The scientist can’t do that… That’s why there is need for… some third party who will intervene, and ensure some equity? Scientists… are best in the laboratories… There must be some form of third part to handle this.”* (CAB, P5)

#### Government and the Ministry of Health.

Many looked to the state to underwrite infrastructure and distribute products, while a substantial current of distrust qualified that hope, which has direct design implications for any government-custodian model.

*“I think the Federal Ministry of Health (in Nigeria) has a role to play in the distribution of drugs developed as a result of the research.”* (FGD, R12)

*“It is supposed to be our government [who ensures the medications… distribution], but our government has many challenges… There are too many bad people in our government.”* (CAB, P3)

#### International bodies.

Some looked above the national level to international health institutions for capacity and legitimacy.

*“The World Health Organization can help to build laboratories like what is obtained in advanced countries to aid local production of such drugs.”* (FGD, R11)

#### Repositories and funders, through binding instruments.

Bioethicists described the machinery that already governs sample distribution and could carry benefit-sharing obligations: material transfer agreements, program-officer vetting, and mandatory data return.

*“no one is allowed to resell the samples… any data they collect… has to go to dbGaP. So, whatever they produce is given back.”* (BE, P5)

Local researchers and the CAB recurred as proximal guarantors and trust anchors across groups, consistent with the sustained-engagement finding of the previous study [1]. No single actor was trusted to do everything; the data point toward a layered arrangement rather than a single custodian.

### The global reach of fair return

Several participants did not confine the beneficiaries to their own community. They extended the logic of fair return to other under-served populations, proposing that products of the research reach low-income countries generally. This widening reflects a moral framing in which the donor community is a representative contributor to a shared human resource, and it aligns benefit sharing with a global-health-equity agenda rather than a narrowly local claim.

*“I think drugs produced through research should be brought to less developed countries so that it can be sold.”* (FGD, R2)

This outward-facing view coexisted with, rather than displaced, the expectation of concrete local return. Participants held both that their own community should benefit tangibly and that the broader good served by the research should reach others like them, a combination that any distribution mechanism must be able to honor simultaneously.

### Timing, transparency, and feasibility

Two conditions were treated as necessary for any of this to be meaningful. First, terms should be agreed at the outset and made explicit in the consent, rather than negotiated after value has been created.

*“we probably need to articulate it in these consent forms so that people can understand and so that we can be clear, with participants… the participants need to know that.”* (BE, P2)

Second, feasibility must be respected. Bioethicists were candid that good intentions collide with funder rules and the risk of over-promising, and that a requirement to give back is hard to specify and enforce.

*“there’s a limit to how much we could do because of just the bureaucracy… we ran into all these issues with the NIH rules, and what you can and cannot give grant funds for… we wanted to make sure that we weren’t making promises to the community that we weren’t able to keep.”* (BE, P3)

*“there’s a part of me that says, yeah, if they can do something to give back to a community, then do it. Which is why I said justification rather than a requirement… where do you draw the line in terms of giving back for commercial entities?”* (BE, P2)

The cross-stakeholder pattern is therefore complementary rather than contradictory. Community and CAB participants supplied the content of fair return (subsidized medicines, infrastructure, a share of value) and named candidate guarantors; bioethicists supplied the constraints (funder rules, anonymity, enforcement, consent articulation) and the instruments (MTAs, data return) through which return could actually travel. An implementable model must join the two.

### A benefit-sharing implementation blueprint

We synthesize the findings into three components: a tiered benefit-bundle menu, an accountable-guarantor model, and contractual insertion points. Table 1 maps each benefit bundle to a delivery mechanism, an accountable guarantor, the principal feasibility constraint, and the illustrative evidence from which it is drawn.

### A tiered benefit-bundle menu

The bundles are not equivalent, and a workable model should not present them as an undifferentiated wish list. We propose a minimum set that any reuse of YRI-type samples for iPSC research should deliver, comprising recognition of the contributing community, sustained transparent reporting, and a concrete capacity-building or access commitment proportionate to the project. Beyond this floor, optional add-ons, subsidized product access, infrastructure, and, where commercial value is realized, a proportional share, are negotiated at project start with the community’s representatives. Tiering matters because it separates what should be guaranteed regardless of commercial success from what is contingent on it, which is precisely the compensation-versus-benefit distinction participants drew [4, 5].

### An accountable-guarantor model

No single actor commanded universal trust of our participants, so we propose a layered guarantor architecture rather than a single custodian. Local researchers and the CAB are the proximal anchor, holding contextual legitimacy and continuity with the community. A national layer (an appropriate health-system or ethics authority) carries statutory reach for product distribution, subject to the safeguards that participants’ distrust of government makes necessary. An independent fiduciary, potentially convened with an international body, holds and disburses ring-fenced funds and any proportional share, insulating benefit delivery from both the research team and political capture. Crucially, and consistent with community requests in our previous study, the model includes a complaint and verification channel that does not run through the research team, so that a community can contest delivery without jeopardizing the relationship it depends on [1, 13]. Escalation runs from proximal to national to fiduciary layers when a benefit is not delivered.

### Contractual insertion points

The instruments that already govern sample distribution can carry benefit-sharing obligations without new bureaucracy. Material transfer agreements can incorporate, alongside existing prohibitions on resale and on unauthorized uses, an explicit benefit-sharing or product-access commitment and, where relevant, a royalty clause directed to a community fund; the mandatory return of derived data to public repositories already models the principle that “whatever they produce is given back” [1]. Consent and community-facing documents should articulate the possibility of commercialization plainly, as bioethicists urged, so that expectations are set at entry rather than contested later. Periodic community reporting, already practiced by the repository, becomes the audit trail against which benefit delivery is checked.

## Discussion

In this study of stakeholder perspectives on benefit sharing for iPSC research using legacy YRI samples, we found that participants did not need to be persuaded that benefit sharing matters, but that they lacked mechanisms. Our contribution is to convert this endorsement into an architecture that can be implemented, comprising a tiered bundle menu that distinguishes guaranteed from contingent returns, a layered guarantor model that matches each benefit to an actor with the legitimacy and competence to deliver it, and contractual insertion points that route obligations through instruments that already exist. This treats benefit sharing for LMIC samples as a problem of implementation rather than of principle.

The normative grounding of these expectations is reciprocity and not charity. Participants insisted that researchers should not engage people in research and then forget about them, and bioethicists rejected the position that a contribution to the general good of science is a sufficient return in an LMIC community context.

### Comparison with existing frameworks

Benefit sharing for genomic resources is already addressed by several frameworks, and our architecture is intended to complement rather than replace them. The Human Genome Organization Statement on Benefit-Sharing established that benefit is owed to contributing communities, and grounded that obligation in common heritage, distributive justice and solidarity [6]. The Nagoya Protocol established that benefits arising from the utilization of genetic resources should be shared fairly and equitably with the providers of those resources and created state-level obligations for access and benefit sharing [8]. The CIOMS guidelines require that research responds to the health needs of the communities in which it is conducted and that benefits be made available to them [7]. Communitarian accounts derive reciprocity, solidarity and accountability as governing values for African genomics, and provide the normative grounding on which claims to fair return rest [4]. Calls for global biobank governance identify the cross-border character of the problem and the need for governance frameworks that distribute risks and benefits fairly between stakeholders [13].

These frameworks establish that benefit sharing is owed and why it is owed. They do not specify which benefits count as fair for a given resource, who holds the obligation when the samples are anonymized and the products are commercialized abroad, or through which instrument an obligation could be enforced. This is the gap our participants encountered. Genomics researchers in sub-Saharan Africa support benefit sharing on grounds of fairness, solidarity and reciprocity, but have little awareness of what it entails and lack clarity about how it could be implemented [5]. Our findings show the same pattern among the source community, its advisory board and the bioethicists who steward the resource, all of whom endorsed benefit sharing and none of whom could name a mechanism that would deliver it.

Two adjacent bodies of work come closer to implementation. Broad consent reframed as consent to governance, and the account of consent as an entrustment, both relocate the ethical question from a single act of permission to an ongoing relationship of stewardship, and both imply that the community retains a claim over time [14–16]. They specify the character of the relationship but not the content of the return or the identity of the guarantor. Our architecture takes the next step. It specifies what is owed through a tiered benefit-bundle menu that separates a guaranteed minimum from returns contingent on commercial success; it specifies who owes it through a layered guarantor model in which local researchers and the community advisory board are the proximal anchor, a national authority carries statutory reach and an independent fiduciary holds ring-fenced funds; and it specifies how the obligation travels, through clauses inserted into material transfer agreements, data-return requirements and product-access commitments (Table 1). Table 2 summarizes what each framework establishes, what it leaves unspecified and what our architecture adds.

Considered within global health, the case exemplifies how commercial and governance determinants, rather than clinical factors alone, shape who benefits from biomedical innovation. LMIC samples sit at the intersection of intellectual property, cross-border data and material flows, and unequal state capacity; treating benefit sharing as a purely local, post hoc courtesy leaves those determinants untouched. Building return into portable instruments and into an accountable intermediary is a way of acting on the structural, not merely the interpersonal, level, which is where durable equity gains are made [9, 13]. The participants who extended fair return to other under-served countries were, in effect, articulating this structural view from below.

Two features distinguish the case of use of the YRI samples for iPSC case. First, anonymization converts benefit sharing from an individual to a communal obligation. Because donors cannot be re-identified, the community becomes the beneficiary and the counterparty for negotiating and receiving benefit, which corresponds to entrustment and persistence-based accounts of governance in which the persisting community, rather than the dispersed individual, holds standing over time [15, 17, 16]. Second, iPSC lines can travel far into global commercial pipelines, so benefit delivery must be built into portable instruments such as material transfer agreements, data return requirements and product access clauses, which can survive distance and intermediaries, rather than into local goodwill. Community preferences and bioethicist knowledge of feasibility must therefore be joined, because the community specifies fair content and the governance machinery specifies deliverable form.

The separation of compensation from benefit has concrete policy implications. Treating a one-time transport reimbursement or participation payment as discharging the obligation is, on our participants’ account, no longer acceptable in an LMIC community context; benefit is ongoing, communal, and proportionate to value created. Encoding this distinction, guaranteeing a floor while making a share contingent on commercial success, protects communities from extraction without exposing researchers to promises they cannot keep, a concern that the bioethicists in this study raised pointedly about funder rules and enforceability.

The justification-versus-requirement question that one bioethicist posed is, in our reading, resolved by tiering rather than by choosing a side. A minimum bundle, recognition, transparent reporting, and a proportionate capacity or access commitment, can be made a requirement because it is bounded and largely within researchers and repositories control; a proportional share of commercial value is better framed as a justified, negotiated contingency, because its magnitude and timing depend on outcomes no one can guarantee at project start. Encoding the two differently keeps the requirement enforceable and the contingency honest and avoids both empty promises and the opposite failure of treating a token payment as sufficient.

The design of intermediaries must account for distrust that was widely expressed by community members. Support for government custodianship was consistently qualified by skepticism about governance. This argues against a pure state custodian model and in favor of an independent fiduciary anchored locally by the CAB and, where useful, convened with an international body such as the World Health Organization, consistent with calls for global biobank governance and for equitable access frameworks in African genomics [9, 13].

For iPSC specifically, benefit sharing cannot be separated from use limits. Participants who welcomed health applications remained wary of reproductive and other contested uses, and repositories already encode such limits in their agreements; a credible benefit-sharing regime sits alongside, and is reinforced by, the boundary-setting that stem-cell oversight requires [3, 10]. The two together express a single message from the community: use our samples for good, treat us as partners, and let us share in whatever profit results.

Our study has several limitations. This is a single community with a strong, possibly atypical relationship of trust with the research team. And the team that conducted this study includes members of the original HapMap study team. The design may therefore overstate optimism about deliverability of the proposals. Secondly, the proposed architecture is a blueprint rather than a costed or piloted mechanism. The most important risk in disseminating this work is expectation inflation. The benefits described are stakeholder preferences, not commitments by any sponsor, institution, or company, and we have been explicit on this point throughout. Future work should cost the minimum bundle, pilot the fiduciary and complaint mechanisms with the community, and test the model in settings where trust in the research team is weaker. The study is qualitative and was not designed to estimate prevalence of preferences.

The study also has two risks that deserve explicit management. The first is expectation inflation, the danger that describing desirable benefits is heard and interpreted as promising them. We have addressed this by labeling all benefits as preferences and by tiering guaranteed from contingent returns, but investigators using this architecture should communicate the distinction to communities directly. The second is a securitizing drift in which oversight and enforcement language frames the donor community, or requesting scientists, as suspect. Benefit sharing should be built as reciprocity and accountability, an expression of partnership, rather than as surveillance. The guarantor and complaint mechanisms exist to protect the community interests, not to police its trust. Framed this way, the model complements rather than contradicts the sustained-engagement and trust-building findings that we previously reported [1], and it operationalizes the reciprocity that communitarian accounts place at the center of just genomics governance [4].

Finally, benefit sharing is one element of a larger governance question about whether legacy samples still represent, and still honor, the people and community from which they came as they travel into new uses. Fair return is the material expression of that continuing relationship; use limits, re-engagement, and transparent reporting are its other expressions [17]. Treating benefit sharing in isolation risks reducing a relational obligation to a transaction, which is precisely the reduction our participants resisted when they distinguished ongoing communal benefit from one-time payment.

## Conclusions

Stakeholders connected to the YRI HapMap samples accept that iPSC research may be commercialized but treat fair return to the contributing community as a condition of legitimacy rather than an optional courtesy. They distinguish one-time compensation from ongoing, communal benefit; they favor subsidized product access, infrastructure, capacity building, recognition, and, for some, a proportional share; and they look for an accountable intermediary that is neither the research team alone nor an untrusted government acting alone. Translating these preferences into a tiered benefit-bundle menu, a layered and independently auditable guarantor model, and benefit-sharing clauses inserted into existing transfer and consent instruments provides a feasible path from principled support to deliverable practice, one that keeps benefit sharing explicit, communal, and proportionate while guarding against promises that cannot be kept.

## Figures and Tables

**Table 1:** Benefit-sharing options matrix for iPSC research using LMIC samples.

Benefit bundle	Delivery mechanism	Accountable guarantor	Principal feasibility constraint	Illustrative evidence
Subsidized product/medicine access	Research- or product-funded subsidy; tiered pricing to donor and participating sites	National health system / Ministry of Health; product-access clause enforced by repository/funder	Downstream, distant products; enforcement across borders and time	“at a subsidized rate… funded by the research” (FGD, R4)
Community infrastructure & development	Ring-fenced community fund; facility or laboratory build	Independent third-party fiduciary; local researchers + CAB	Funder rules on allowable costs; sustainability	“the hall donated to the Aba-Alamu community” (FGD, R9)
Capacity building & local co-investigation	Training, co-authorship, local reprogramming capacity, recognition on the resource	Consortium/repository; funding agency	Time horizon; measurement of “capacity”	“recognized that they’re on the map… capacity building” (BE, P1)
Proportional revenue / royalty share	Weighted percentage of net commercial return into community fund	Independent fiduciary with escrow; MTA royalty clause	Valuation; anonymity; legal complexity	“this source will get a 2.5%, a 5%… weight it… and build that” (CAB, P5)
Recognition & governance participation	Named acknowledgment; community seat in oversight/reporting	CAB; repository governance	Tokenism risk; contextual knowledge	“indicate where the samples come from… benefits come back” (FGD, R13)
Individual compensation	Direct payment (only where identifiable)	Not available under anonymization; redirected to community	Anonymized samples preclude individual records	“if not, their community should be considered” (FGD, R12)

CAB, community advisory board member; FGD, focus group participant; BE, bioethicist; MTA, material transfer agreement. Quotations are illustrative and verbatim; benefits listed are stakeholder preferences, not commitments.

**Table 2:** Comparison of the proposed benefit-sharing architecture with existing frameworks.

Framework	What it establishes	What it leaves unspecified	What our architecture adds
HUGO Statement on Benefit-Sharing [6]	That benefit is owed to contributing communities, grounded in common heritage, distributive justice and solidarity; recognition and capacity building count as benefit	Which benefits are owed for a given resource; who delivers them; how they are enforced	A tiered bundle menu that makes recognition and capacity building the guaranteed minimum rather than aspirations
Nagoya Protocol [8]	State-level obligations for access and fair, equitable sharing of benefits from genetic resources	Communal delivery where donors are anonymized; mechanisms where value arises far downstream and abroad	An independent fiduciary anchored locally, for settings where state custodianship alone is not trusted
CIOMS guidelines [7]	That research must respond to community health needs and that benefits be made available to them	The content of the benefit; the guarantor; the timing of agreement	Product-access and subsidy clauses, with terms agreed at project start and stated in the consent
Consent to governance; entrustment [15, 14]	That permission is an ongoing relationship of stewardship rather than a single act, and that the community retains a claim over time	What is actually returned, by whom, and through which instrument	Specification of content, guarantor and instrument, so that stewardship becomes auditable
Ubuntu communitarian governance [4]	Reciprocity, solidarity and accountability as governing values for African genomics	Translation of values into deliverable, enforceable mechanisms	An implementation layer that operationalizes reciprocity without reducing it to a transaction
Global biobank governance [13]	The cross-border character of the problem and the need for fair distribution of risks and benefits	The delivery architecture at the level of a single donor community	Contractual insertion points that travel with the sample across borders and intermediaries
Empirical benefit-sharing studies [5]	That African genomics researchers support benefit sharing but lack awareness of what it entails and how to implement it	Mechanisms that would resolve the implementation gap the studies document	A blueprint derived from the stakeholders themselves, including the source community

HUGO, Human Genome Organization; CIOMS, Council for International Organizations of Medical Sciences.

## Data Availability

De-identified excerpts supporting the findings are presented within the article. The full qualitative dataset is not publicly available in order to protect participant and community confidentiality but may be available from the corresponding author on reasonable request, subject to the governance conditions described.
